# Mental health outcomes before psychotropic medications: a retrospective case series of one state hospital records from 1945 to 1954

**DOI:** 10.1186/s12913-023-09235-8

**Published:** 2023-03-15

**Authors:** Ann Chapleau, Jennifer Harrison, Stephen Love, David Sherman

**Affiliations:** 1 Western Michigan University, Kalamazoo, USA; 2 State Hospital Administration, Michigan Department of Health and Human Services, Lansing, USA

**Keywords:** Mental health outcomes, Psychosocial rehabilitation, Historical research, State psychiatric hospitals, Retrospective case series, Discharge status

## Abstract

**Background:**

Current outcomes for mental illness are widely regarded as poor. Since the introduction of psychotropic medications in the mid 1950’s, previous psychosocial practices were minimized in favor of medication focused treatment. The majority of large U.S. state hospitals have closed with records destroyed or in storage, inaccessible to researchers. This creates barriers to studying and comparing outcomes before and after this shift in treatment practices.

**Aims:**

The study aim was to examine discharge outcomes in relation to length of stay and diagnosis in one U.S. state hospital.

**Methods:**

This case series study examined 5618 medical records of participants admitted to one state hospital from 1945 to 1954, the decade prior to adoption of psychotropic medications.

**Results:**

Of the 3332 individuals who left the facility, over half (59.87%) of first episode hospitalizations were discharged within 1 year, and 16.95% were hospitalized for more than 5 years. 46.17% of all admissions were discharged from hospital with no readmission. The most common diagnoses included schizophrenia, other forms of psychosis, and alcoholism. In the decade before the introduction of psychotropic medications, participants were often admitted for a single episode and returned to their homes within several years.

**Conclusions:**

Although limited to one site, findings suggest that discharge outcomes prior to psychotropic medication as a primary treatment for mental illness may be more positive than previously understood.

## Introduction

Standard mental healthcare practices in the first half of the twentieth century included insulin shock therapy, sensory deprivation, and lobotomies, as well as anticonvulsant and barbiturate drugs, none resulting in significant improvements in psychiatric symptoms [1]. By the 1930s, practices at state psychiatric hospitals included occupation-based activities such as vocational training, farming chores, food preparation, laundry and other daily tasks, recreational activities, sensory-based interventions such as music, massage, and hydrotherapy, and extensive social services including family care (placement in a private home with non-relatives) for community re-entry [2, 3]. With the introduction of the first psychotropic medication, chlorpromazine, known as Thorazine, in the mid-1950s, there were high expectations of the new “miracle drugs” that would dramatically improve mental health outcomes [4]. At the time, there were references to the ‘euphoric quietude’ brought about by psychotropic medication chlorpromazine or Thorazine and the hope that medication would fundamentally change psychiatric outcomes [5, 6]. Following approval from the U.S. Food and Drug Administration in 1954, treatment shifted to primarily supporting a medication-based model within state psychiatric hospitals, with fewer resources available for psychosocial and occupation-based care [7, 8]. This trend has continued over the intervening decades, even as many state psychiatric hospitals were shut-down and more services for people with serious mental illness (SMI) were provided within the community [9].

As pharmacological treatments became one of the primary practices within mental health care, and the pharmaceutical industry gained influence, research investigating the outcomes of psychosocial treatments fell out of political interest. The National Institute of Mental Health’s funding for research decreased by 5% each year from 1965 until 1972, for a total decrease of 30% [10, 11].

Despite the plethora of pharmacological treatments today, and the booming pharmaceutical industry, adult disability rates have increased from 1 in every 468 adults in 1955 to 1 in every 59 adults in 2013 [12]. Approximately 2.5 – 3.5 million people who have serious mental illness live in poverty with poor and unstable housing [13]. The incarceration rate for those with mental illness has more than tripled since the 1960s, with approximately 2.2 million currently in prisons and jails which do not have the resources to provide mental healthcare [14]. Moreover, treatment stays are lengthening. In 2015, the average median length of stay for patients admitted to state-run psychiatric hospitals was 75 days [15] which is markedly above findings from a 2006 study, which excluded long-term care facilities and government hospitals, that found the average length of stay to be 10 ± 3 days [16].

To make improvements in mental healthcare practices, it can be useful to understand the history of mental healthcare in the U.S., including discharge outcomes prior to this shift in resources and practices that began in the mid 1950’s [10, 17, 18]. Although much of this data has been absent in the literature, it is possible to preserve and digitize remaining records to allow study comparing outcomes prior to the introduction of psychotropic medication, which marked the drastic shift in resources and practices away from psychosocial rehabilitation to current medication-focused treatment that is regarded as best practice.

### Background

Previous studies investigating outcomes for individuals at state psychiatric hospitals are sparse and provide varying definitions and estimates of discharge status and lengths of stay [19]. These studies, however, provide insight into the numbers of those admitted into state psychiatric hospitals, what diagnoses individuals were given, and length of stay. In one multi-state study, the mean length of stay for patients with schizophrenia in the 1950s was over 13 years [20, 21], but less than 50% of patients were hospitalized for 8 years, bringing the median length of stay for all diagnoses to under 8 years [21]. Other studies highlight a contrasting image in showing successful discharge of patients over time. Rates of discharge from first admissions dramatically increased from 54.9% of patients with a diagnosis of schizophrenia discharged within ten years of admission between 1913 and 22 to 72.5% of patients with a schizophrenia diagnosis discharged within ten years of admission between 1943 and 52 [22]. This increase in discharge did not result in a corresponding increase in readmission rates, meaning that a greater percentage of people were discharged to the community and never re-hospitalized [22]. This data is consistent with other studies of psychiatric outcomes during the time period prior to the introduction of psychotropic medications in the mid-1950’s. At a state psychiatric hospital in New York in the mid-1940s, 44.27% of patients diagnosed with schizophrenia were never readmitted and 49.9% were not in the hospital at follow-up 5 years after first admission [23]. In the Ohio State Mental Hospital between 1948 and 1952, Locke [24] observed that within 5 years of first admission, 70% (*n* = 5781) of all patients hospitalized were discharged. Furthermore, 40% of individuals diagnosed with schizophrenia were discharged within 6 months of first admission and 70% of individuals diagnosed with schizophrenia were discharged within 2 years of their first admission [24]. Variables such as marriage, employment, education, and age were all positive predictors of discharge from the hospital [24].

A potential reason for the vast differences in these discharge rates is the lack of a uniform definition of what constitutes discharge [24]. For many patients diagnosed with schizophrenia, the actual time spent in the hospital is much less than indicated by the admission and discharge date [24]. In many state psychiatric hospitals, patients left the hospital and returned to the community in order to ensure they had the skills and resources needed to succeed or return to the hospital if their condition worsened. A current issue faced when studying historical mental health outcomes is the varied approaches that researchers have taken in determining what constitutes a discharge. Each hospital and facility had a unique system of documenting the medical record of admitted patients [25]. Literature that combines data from different hospitals fails to recognize many of the unique characteristics that are important to document along with discharge. For example, a multi-state analysis of first admissions of patients within the year of 1954 provided only discharge status, which was limited to whether the patient died, was released, or retained [26]. This leaves much unknown regarding the condition of the patient at discharge [26].

In a review of first admissions to psychiatric hospital studies, [19] provided statistical reporting from literature that examined outcomes of patients diagnosed with schizophrenia. The discharge data provided was limited to death, discharge, or still in the hospital. In addition, the authors included that their data on discharge was only an estimation of what happened. Whether the patient was improved or unimproved, discharged to the care of family or independently, and if the discharge was planned or via escape is unknown to the reader. Discharge circumstances and environment are crucial in predicting successful outcomes from mental illness and the existing literature and available data provide limited information on these areas [27].

The primary goal of the researchers was to capitalize on a rare and unique opportunity to study and compare discharge outcomes before and after the shift to primarily medical model care in the mid-1950s, at one U.S. state psychiatric hospital that has continually provided services since 1859. The aims of this first study were to explore the various types of discharge between 1945 and 1954 and to systematically categorize each type of discharge to enable further study. An additional goal of this study was to examine different discharge outcome variables in relation to length of stay and re-hospitalizations of individuals between 1945 and 1954 and discharged any time after first admission.

## Methodology

This retrospective case series study examined 5618 unique medical records of participants with a first time (episode) admission at one Midwestern state psychiatric hospital from 1945 through 1954. This period of time represents the decade just prior to the introduction of psychotropic medications as the primary treatment for mental illness.

### Procedure

#### Data sources

**Fig. 1:**
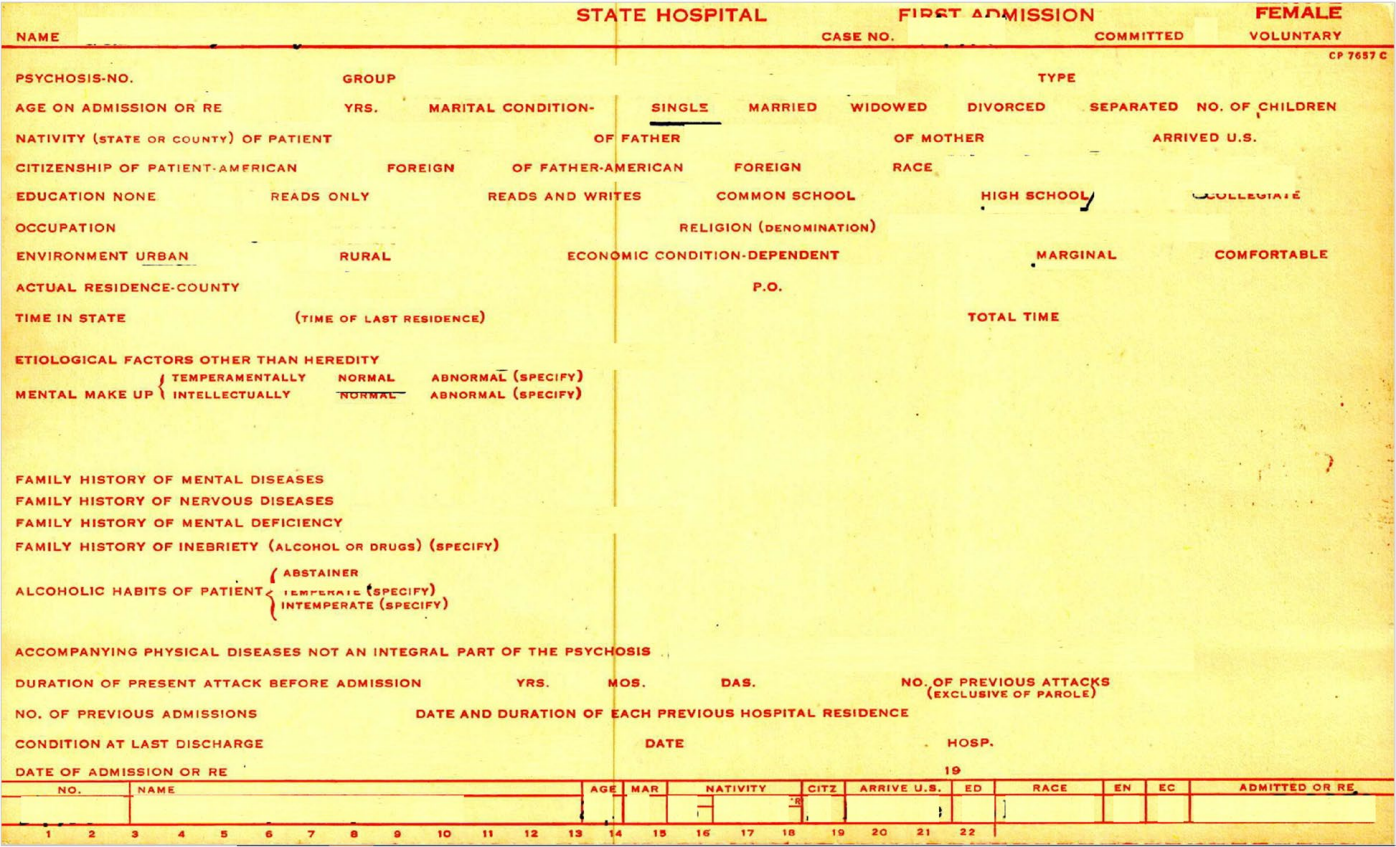
Data Source from Medical Index Cards. *Note*. Above is a figure of the index card from which data originated from, with all identifying information redacted

Physical (non-digitized) medical records with episode information about each patient were reviewed with data entered into a spreadsheet on a password protected computer. An example of the medical records can be seen on Fig. 1. Data were de-identified using the Safe Harbor Method [28, 29]. Upon de-identification, data were stored on an encrypted flash drive throughout the study.

### Variables

In order to uncover the meaning of the various discharge types, it was necessary to examine the records to uncover how each designated status was used. Each medical record listed the type of leave, be it discharge or otherwise, discharge status and the date that was it given. This system allowed the hospital to track participants each time they would enter and exit the hospital. Variables as shown in Table 1 included in this sample were: age at first admission, gender, diagnosis, and race.

**Table 1:** Patient demographics and diagnosis

*Sample demographics*			
	*n*	*n* Died in Hospital	*n* Discharged alive
**Gender**			
Male	2680	*	*
Female	2929	*	*
Missing	9	*	*
Total	**5618**	*	*
**Age**			
0-10	6	0	6
11-20	246	12	234
21-30	731	47	684
31-40	856	71	785
41-50	827	116	711
51-60	737	185	552
61-70	737	338	399
71-80	910	680	230
81-90	431	366	65
91-100	27	23	4
Missing	110	23	4
Total	**5618**	**2286**	**3332**
**Race**			
African	133	44	89
Dutch	291	120	171
English	271	148	123
German	520	220	300
Irish	177	78	99
Mixed	3112	924	2188
Negro	207	34	173
Scandinavian	91	31	60
Slavonic	256	66	190
Unknown	199	85	114
Other	341	97	244
Missing	20	9	11
Total	**5618**	**2286**	**3332**
**Diagnosis**			
Psychosis with Cerebral Arteriosclerosis	790	573	217
Schizophrenia Paranoid Type	641	78	563
Senile Psychosis Simple Deterioration	384	319	65
Schizophrenia Catatonic Type	295	16	279
Schizophrenia Hebephrenic Type	174	30	144
Schizophrenia Reaction Paranoid Type	135	13	122
Psychosis with Syphilis Menigo Encephalitis	105	50	55
Schizophrenia Reaction Catatonic Type	91	7	84
Involutional Psychosis Melancholia	83	13	70
Undiagnosed Psychosis	82	30	52
Without Psychosis Chronic Alcoholism	82	0	82
Other	2755	727	2028
Missing	1	0	1
Total	**5618**	**2286**	**3332**

*Note*. Racial identities reflect categorization and verbiage from the time period, not current reflection of racial and ethnic understanding

*Death while in the hospital by gender was not collected or available

### Condition on discharge status

Of those discharged into the community, a condition on discharge [30]. was recorded. The condition upon discharge statuses were observed and their corresponding definitions can be found in Table 2. Patients were often placed on long term leave and formally discharged after several months to years at which point they were given a condition on discharge status. Thus, the researchers found the data difficult to accurately represent condition upon discharge as the condition was often assigned months after initial leave from hospital [23]. For that reason, we analyzed the actual physical index cards and determined four types: community discharge, death, escape, or transfer. These categories more accurately represent how discharge is defined and determined today.

**Table 2:** Condition upon discharge codes

Condition	Meaning & definition
Esc	Escape from the hospital. Was counted as a discharge from the hospital if the patient had been escaped for more than 1 week
Out	Bookkeeping notation that marked a temporary leave from the hospital that would be followed by a soon return (i.e., Return)
Leave	Bookkeeping notation that marked a temporary leave from the hospital that would be followed by a soon return (i.e., Return)
^a^ To FC	To family care, a discharge from the hospital to a type of rudimentary adult foster care. Placement of psychiatric patients in the community with non-related family
Return/Ret	Not considered a readmission if it was preceded by an Out or Leave. If the Return/Ret was preceded by a discharge, it was considered a readmission to the hospital
Conv St Par	Convalescent status, a discharge from the hospital Parole, a discharge from the hospital
Direct Disch	Direct discharge was a discharge from the hospital
Disc from Par	Discharge from Parole, a book keeping notation, not an actual discharge from the hospital
Ret Soundness of mind	A legal restoration of sanity, not a discharge from the hospital
Trans to	Patient transferred to another facility, a discharge from the hospital
Death	A discharge from the hospital. Condition and cause of death was listed
Imp	Improved, documented and observable improvement upon discharge
Unimp	Unimproved, no documented improvement

*Note.* ^a^ Family care (to FC), as it was used at this point in history, generally refers to the placement of psychiatric patients in the community with a non-related family (Tuntiya, 2006). This practice originated in Gheel, Belgium and was brought into use by this hospital in the late 1930s (Mallon, 1958) with roughly 20 family homes established by 1942 [7]

Inclusion criteria for the study included all medical records of participants hospitalized for the first time beginning January 1, 1945 through December 31, 1954. Individuals who were first admitted before the dates of the study and were readmitted during studies time were excluded from analysis. Additionally, participants who were admitted outside of the study dates were excluded from analysis. Data entry into the database began in March 2015 until March 2021.

### Planned analysis

After coding the initial sample, data was imported into SPSS, version 27, for univariate statistical analysis. Data was cleaned and transformed extensively using R. Packages used included dplyr, base, stringr, and forcats. After the process of cleaning and transforming, data was coded. Univariate analysis was then completed in order to determine frequencies and distributions of the samples’ various demographic variables. Additional univariate analysis was conducted to create mean and median for each subcategories of diagnosis and their respective length of stay associated with their admission.

## Results

The data collection consisted of 5618 unique medical records from participants that had been admitted to the hospital beginning on January 1, 1945 and ending December 31, 1954. The entire sample is comprised of 2680 male and 2929 female participants. Nine cases had missing data for gender. The mean age for the 5508 cases with data related to age (110 missing cases) upon first admission was observed being 52.3 (SD = 20.2). Of the 5618 admissions, 2286 (40.69%) individuals died while in the hospital, and 3332 (59.31%) were discharged alive and never readmitted to the same hospital. The median length of stay for people who were discharged from the hospital was 230 days.

It is noteworthy that such a high percentage of individuals, slightly over 40%, died during their hospital stay. This can be understood by a brief description of the nature of state psychiatric hospitals in the United States in the first half of the twentieth century. Psychiatric hospitals were utilized for much more than short term psychiatric stabilization than they are currently. Before the introduction of skilled nursing homes, psychiatric hospitals were often used for the treatment of dementia, post-stroke care, and other cardiovascular conditions. This can be seen for example in the 790 participants with the diagnosis of Psychosis with Cerebral Arteriosclerosis, of which 573 (73%) died in the hospital. Similarly, of the 384 participants diagnosed with Senile Psychosis Simple Deterioration, 319 (83%) died in the hospital. In addition, patients with syphilis or tuberculosis were treated in psychiatric hospitals. Of the 105 patients diagnosed with Psychosis with Syphilis Menigo Encephalitis, 50 died (47.62%) in the hospital. It is unclear how many of the hospitalized patients also had tuberculosis as a co-occurring disorder. Cause of death was not present in the medical record. The entire sample’s *n* and frequencies of death can be observed in Table 1.

The sample included a total of 48 categories of racial identity. The most occurring race observed in the sample was “Mixed,” *n* = 3112 followed by second most occurring race was “German,” *n* = 520. The top ten racial identities and frequencies included in the sample can be observed in Table 1.

### Condition upon discharge status

There was a total of 11 subcategories of condition upon discharge statuses in the full sample (*n* = 3332) as shown in Table 3. Aside from death, the most frequent status was "improved", comprising 1750 (31.1%) individuals. The remaining condition upon discharge statuses observed in the full sample can be observed in Table 3.

**Table 3:** Observed condition upon discharge

Condition	*n*
Death	2286
Improved	1750
Missing	616
Unimproved	301
Without Psychosis	175
Transfer	165
Paroled	99
Escaped	90
Recovered	64
Convalescence	46
To Family Care	26
Total	5618

### Length of stay for community discharge status and diagnosis

Over three thousand (*n* = 3332) of first episode admissions were discharged into the community. Of these participants with a discharge, total *n* = 1995 (59.87%) of participants were discharged within their first year of admission. Admissions longer than 5 years include a total *n* = 565 (16.95%).

Due to first episode length of stay being non-normally distributed, both the mean and median length of stay for each diagnosis is reported. For example, the mean length of stay for Schizophrenia, Catatonic Type is 1377.7 days where the median length of stay is 234 days. Table 4 provides a complete breakdown of the mean and median length of stay separated by diagnosis for patients discharged from the hospital into the community.

**Table 4:** Length of stay by diagnosis for patients who were discharged from hospital

Diagnosis	N	Mean LOS	Median LOS
Psychosis with Cerebral Arteriosclerosis	153	503.69	164
Schizophrenia Reaction Paranoid Type	110	1441.91	575.5
Senile Psychosis Simple Deterioration	25	683.36	195
Schizophrenia Catatonic Type	257	1377.7	234
Schizophrenia Hebephrenic Type	114	3794.95	1983.5
Schizophrenia Paranoid Type	492	1895.68	427
Psychosis with Syphilis Menigo Encephalitis	51	1646.20	377
Schizophrenia Reaction Catatonic Type	79	1532.63	454
Involutional Psychosis Melancholia	66	714.85	166.5
Undiagnosed Psychosis	46	276.2	123.5
Without Psychosis Chronic Alcoholism	80	162.94	95
Other	1858	1074.32	194
Missing	1	427	427
Total	3332	1274	230

*Note.* Length of Stay is represented by the number of days after first admission until discharge

## Discussion

The findings of this study provide needed data about the outcomes of participants admitted to one state psychiatric hospital before the introduction of psychotropic medication. The results demonstrate that in the decade before the introduction of chlorpromazine, there exists ample evidence that participants were routinely admitted for only a single episode. The vast majority of participants were discharged into the community following inpatient psychiatric service within several years, with the median length of stay being 230 days. Over half of individuals were discharged within 1 year of admission to the hospital. Only 19.01% had a length of stay over 5 years, mirroring findings within previous literature [24]. These results do not support the narrative that before the introduction of psychotropic medications, the majority of individuals who were hospitalized for psychiatric care had lengths of stay that lasted many years or even decades [4–6].

Additional findings of note include those individuals with a diagnosis of the different schizophrenia subtypes, who had the longest mean lengths of stay. By contrast, individuals with a diagnosis of alcoholism had a much shorter length of stay. This may be related to the culturally bound constructs surrounding individuals with substance use disorders, and whether they are considered mental health conditions or merely poor moral conduct.

This study provides further value by addressing and determining the different type of discharge status and environments participants were released to, which are documented as being crucial when predicting successful psychiatric outcomes [27]. Previous studies have left out the differing types of discharge status which leaves out imperative information on the condition of participants upon discharge [25]. In fact, when the 40.69%% of participants who died in the hospital were excluded from the final analysis of discharge status, the most common discharge status was improved, followed by unimproved, only 301 participants. This speaks to the issue of individuals admitted to psychiatric hospitals with senile conditions and other age-related organic brain disorders being co-mingled within samples at the time.

Many patients who were admitted to state psychiatric hospitals also had multiple diagnoses, often suffering from physical ailments or, experiencing the effects of natural aging. Prior to the 1980s, many patients with dementia or related organic brain disorders were treated in state psychiatric hospitals, whereas after 1980 they were more likely to be treated in skilled nursing facilities [31]. This “lumping together” of psychiatric conditions with physical and cognitive disabilities provides misleading data about both lengths of stay and outcomes such as death. This study was able to separate individuals who died in the hospital from those who were discharged from the hospital. This is important because people who died in the hospital were often admitted for non-psychiatric conditions, such as dementia, tuberculosis, syphilis, and stroke. Findings provide additional context into the nature of psychiatric hospitalization prior to the introduction of Thorazine. Further study is needed to compare psychiatric hospital outcomes in the first half of the twentieth century to today, as diagnoses and discharge are reported differently. This study demonstrates the importance of exploring historical data and posits that outcomes prior to medication as a primary treatment for mental health illness were more positive than was previously understood.

### Limitations

The current study only examined first episode length of stay. This information is provided in the sample but the current study did not analyze the readmission statistics included. Additionally, participants could have been admitted to other inpatient or outpatient psychiatric services after discharge, although there were minimal alternatives at that time.

Another limitation includes the difference between how we currently classify mental health diagnoses and discharge compared to previous classifications [32]. This creates difficulty when attempting to draw conclusions from past to current constructs. A third limitation is the lack of information about cause of death for the 2286 individuals who died in the hospital. Since many of them had non-psychiatric illnesses, their death may have been related to physical illnesses but is unknown.

Due to the lack of clarity on what treatments were being used at the time and their specific effect on participants, it is difficult to draw conclusions that explain these results. Therapies such as psychotherapy, work, and occupational therapy were all treatments that were provided during the time frame of the study [33]. What specifically was responsible for the discharge outcomes is unknown.

### Study implications

Future study of current data is needed in order to examine and explore the relationships that age, race, gender, and education have with both diagnosis and length of stay. Previously, variables including gender, employment status, education level, and age have been recorded as being positive predictors of discharge from psychiatric hospital [24]. Given the richness of data included in this study of over 5000 individuals, these variables can be more closely examined to determine the relationship between demographics and diagnosis, length of stay, and discharge status. Study of the historical data provides a more complete understanding of what psychiatric outcomes were and what they looked like at the time just prior to the shift to psychotropic treatment and deinstitutionalization [10, 17, 18].

An additional area for future study includes comparison of treatment interventions and their related outcomes. Prior to the shift to a pharmacologically-dominated model, typical state hospital treatment included occupational, music, and recreation therapies, sensory-based therapies including massage and hydrotherapy, and extensive social services including family care to provide supports as participants transitioned gradually to the community [3]. The researchers plan to study the detailed historical records that have been preserved at this state psychiatric hospital. Study of this qualitative data will provide a more robust understanding of discharges, lengths of stay, and how they were affected by the psychosocial rehabilitation services that participants received during this period.

## Data Availability

The research data, consisting of protected patient information, cannot be shared publicly due to privacy concerns and restriction by the State of Michigan.

## References

[1] Moncrieff, J. An investigation into the precedents of modern drug treatment in psychiatry. History of Psychiatry, x, 1999:475-490. 10.1177/0957154X9901004004. 10.1177/0957154X9901004004 11624330

[2] Andersen LT, Reed KL (2017). The history of occupational therapy: the first century.

[3] Decker WA (2008). Asylum for the Insane: a History of the Kalamazoo State Hospital.

[4] Ban TA (2007). Fifty years chlorpromazine: a historical perspective. Neuropsychiatr Dis Treat.

[5] Braslow JT, Marder SR (2019). History of psychopharmacology. Annu Rev Clin Psychol.

[6] Swazey JP (1974). Chlorpromazine in psychiatry: a study of therapeutic innovation.

[7] Jacobsen E (1986). The early history of psychotherapeutic drugs. Psychopharmacology.

[8] López-Muñoz F, Alamo C, Cuenca E, Shen WW, Clervoy P, Rubio G (2005). History of the discovery and clinical introduction of chlorpromazine. Ann Clin Psychiatry.

[9] Davis L, Fulginiti A, Kriegel L, Brekke JS (2012). Deinstitutionalization? Where have all the people gone?. Curr Psychiatr Rep.

[10] Horwitz AV, Grob GN (2011). The checkered history of American psychiatric epidemiology. Milbank Q.

[11] Wilson M (1993). *DSM-III* and the transformation of american psychiatry: a history. Am J Psychiatr.

[12] Social Security Administration, Office of Policy, Office of Research, evaluation, and statistics. Annual statistical report on the social security disability insurance program. 2003-13. Baltimore; 2004-14.

[13] Balasuriya L, Buelt E, Tsai J. The never-ending loop: homelessness, psychiatric disorder, and mortality. Psychiatr Times. 2020;37(5) https://www.psychiatrictimes.com/view/never-ending-loop-homelessness-psychiatric-disorder-and-mortality.

[14] Lyon, E. (2019) Imprisoning America’s mentally ill. Prison Legal News. https://www.prisonlegalnews.org/news/2019/feb/4/imprisoning-americas-mentally-ill/

[15] Fuller DA, Sinclair E, Snook J (2016). Released, relapsed, Rehospitalized: length of stay and readmission rates in state hospitals: a comparative state survey.

[16] Lee S, Rothbard AB, Noll EL (2012). Length of inpatient stay of persons with serious mental illness: effects of hospital and regional characteristics. Psychiatr Serv.

[17] Caton CL, Goldstein JM, Serrano O, Bender R (1984). The impact of discharge planning on chronic schizophrenic patients. Psychiatr Serv.

[18] McGrew JH, Wright ER, Pescosolido BA (1999). Closing of a state hospital: an overview and framework for a case study. J Behav Health Serv Res.

[19] Ram R, Bromet EJ, Eaton WW, Pato C, Schwartz JE (1992). The natural course of schizophrenia: a review of first-admission studies. Schizophr Bull.

[20] Brown GW (1960). Length of hospital stay and schizophrenia: a review of statistical studies. Acta Psychiatr Scand.

[21] Kramer M. Long-range studies of mental hospital patients: an important area for research in chronic disease. Milbank Q. 2005;83(4). 10.1111/j.1468-0009.2005.00422.x. 13071686

[22] Israel RH, Johnson NA (1956). Discharge and readmission rates in 4,254 consecutive first admissions of schizophrenia. Am J Psychiatr.

[23] Lehrman NS (1960). A state hospital population five years after admission: a yardstick for evaluative comparison of follow-up studies. Psychiatry Q.

[24] Locke BZ (1962). Outcome of first hospitalization of patients with schizophrenia. Public Health Rep.

[25] Kramer M, Goldstein H, Israel RH, Johnson NA (1955). A historical study of the disposition of first admissions to a state mental hospital.

[26] Pollack E, Person P, Kramer M, Goldstein H (1959). Patterns of retention, release, and death of first dmissions to state mental hospitals.

[27] Schooler NR, Goldberg SC, Boothe H, Cole JO (1967). One year after discharge: community adjustment of schizophrenic patients. Am J Psychiatr.

[28] Garfinkel, S. L. (2015).. De-identification of personal information. National Institute of Standards and Technology 10.6028/nist.ir.8053.

[29] Kayaalp M (2017). Modes of De-identification. AMIA annual symposium proceedings.

[30] United States Census Bureau (1935). Patients in hospitals for mental disease: statistics of mental patients in state hospitals together with brief statistics of mental patients in other hospitals for mental disease, Volume 3. U.S.

[31] Manderscheid RW, Witkin MJ, Rosenstein MJ, Bass RD (1986). The National Reporting Program for mental health statistics: history and findings. Public Health Rep.

[32] Manderscheid RW, Ryff CD, Freeman EJ, McKnight-Eily LR, Dhingra S, Strine TW (2010). Peer reviewed: evolving definitions of mental illness and wellness. Prev Chronic Dis.

[33] Swayze VW (1995). Frontal leukotomy and related psychosurgical procedures in the era before antipsychotics (1935-1954): a historical overview. Am J Psychiatr.

